# A Genetic Circuit Design for Targeted Viral RNA Degradation

**DOI:** 10.3390/bioengineering11010022

**Published:** 2023-12-25

**Authors:** Adebayo J. Bello, Abdulgafar Popoola, Joy Okpuzor, Adaoha E. Ihekwaba-Ndibe, Femi J. Olorunniji

**Affiliations:** 1 School of Pharmacy & Biomolecular Sciences, Liverpool John Moores University, Liverpool L3 3AF, UK; a.j.bello@ljmu.ac.uk (A.J.B.); a.n.popoola@ljmu.ac.uk (A.P.); 2 Department of Biological Sciences, Redeemer’s University, Ede 232101, Osun State, Nigeria; 3 Department of Medical Laboratory Science, Kwara State University, Malete, Ilorin 241102, Kwara State, Nigeria; 4 Department of Cell Biology & Genetics, University of Lagos, Akoka, Lagos 101017, Lagos State, Nigeria; joyokpuzor@yahoo.com; 5 School of Life Sciences, Alison Gingell Building, Coventry University, Coventry CV1 2DS, UK

**Keywords:** genetic circuit, RNA degradation, synthetic biology, RNA-binding protein, RNA virus

## Abstract

Advances in synthetic biology have led to the design of biological parts that can be assembled in different ways to perform specific functions. For example, genetic circuits can be designed to execute specific therapeutic functions, including gene therapy or targeted detection and the destruction of invading viruses. Viral infections are difficult to manage through drug treatment. Due to their high mutation rates and their ability to hijack the host’s ribosomes to make viral proteins, very few therapeutic options are available. One approach to addressing this problem is to disrupt the process of converting viral RNA into proteins, thereby disrupting the mechanism for assembling new viral particles that could infect other cells. This can be done by ensuring precise control over the abundance of viral RNA (vRNA) inside host cells by designing biological circuits to target vRNA for degradation. RNA-binding proteins (RBPs) have become important biological devices in regulating RNA processing. Incorporating naturally upregulated RBPs into a gene circuit could be advantageous because such a circuit could mimic the natural pathway for RNA degradation. This review highlights the process of viral RNA degradation and different approaches to designing genetic circuits. We also provide a customizable template for designing genetic circuits that utilize RBPs as transcription activators for viral RNA degradation, with the overall goal of taking advantage of the natural functions of RBPs in host cells to activate targeted viral RNA degradation.

## 1. Introduction

Viral infections in humans present an ongoing challenge that necessitates constant innovations aimed at developing solutions to respond to new diseases, with the goal of mitigating their impact on our health and the normal functioning of our society. An example is the COVID-19 pandemic, which led to millions of deaths globally and caused an economic downturn in most countries [1]. Like most viruses, one of the challenges encountered in attempts at therapeutic management is the rapid mutation of the SARS-CoV-2 virus [2]. A crucial element of the viral biology process is that viruses depend on host machinery systems to convert their RNA molecules into proteins necessary for their replication to further infect other cells [3,4]. Hence, if the process of converting viral RNA molecules into proteins (translation) can be selectively disrupted, it is possible to reduce the rate at which new viral particles are assembled, hence limiting the ability of the virus to infect other cells. A key requirement for achieving this is establishing a mechanism for the selective degradation of viral RNA.

RNA degradation can be used to regulate gene expression during translation [5]. However, halting the translation of viral RNA into protein may also interfere with the synthesis of necessary host proteins [6]. This is because the ribosome responsible for protein synthesis cannot distinguish between host RNA and foreign RNA. This lack of orthogonality is a recurring issue that often requires designing genetic circuits that operate independently of the host’s pathway to achieve the desired outcome. Therefore, to use targeted RNA degradation as an antiviral strategy, it is essential to have an independent pathway that precisely targets viral RNA and remains orthogonal to the host’s system. This will prevent the random degradation of host RNAs by antivirals and effectively address the challenges presented by this process. One approach to achieve precise RNA regulation is the construction of genetic circuits containing genes/proteins that are assembled orthogonally to express enzymes responsible for viral RNA degradation [7]. There are three classes of RNA-degrading enzymes; the exonucleases that degrade RNA in the 5′ direction, the exonucleases that degrade RNA in the 3′ direction, and the endonucleases that break down internal RNA [8]. A good example of endonucleases is the cas-13 enzyme, which possesses natural RNAse activity against viruses. This enzyme in CRISPR-cas13 systems has been successfully reprogrammed for precise control over RNA degradation [9,10,11]. Abbott et al. [12] developed PAC-MAN (prophylactic antiviral CRISPR in human cells), a CRISPR-cas13 system for degrading influenza A and SARS-CoV-2 viruses. Blanchard et al. [13] also showed that messenger RNA (mRNA)-encoded Cas13a was effective against influenza A and SARS-CoV-2 viruses in mice and hamsters, respectively. In addition, RNA-binding proteins (RBPs), which are upregulated in virus-infected cells, have been shown to play crucial roles in suppressing or influencing host–virus interaction [14], notably zinc finger antiviral protein (ZAP) [15,16] and TRIM25 [17], and these proteins could be repurposed to achieve targeted viral inhibition.

Synthetic biology is a rapidly developing field that could have a significant impact on various aspects of human life. It involves the application of engineering principles to the design and modification of living cells to perform specific functions or create valuable products. Synthetic biologists and biotechnology companies are utilizing basic scientific knowledge to harness the power of nature to tackle challenges related to health, manufacturing, agriculture, and the environment [18,19,20,21]. Some of the applications of synthetic biology in manufacturing include the synthesis of industrial biopolymers or pharmaceutically important compounds. For example, Colloms et al. [22] developed serine integrase recombinational assembly (SIRA) for the rapid assembly of pathways from DNA parts. They used the method to assemble a biosynthetic pathway for lycopene produced in *E. coli.* Awan et al. [23] engineered baker’s yeast (*Saccharomyces cerevisiae*) to produce penicillin by assembling the gene clusters from the natural fungus *Penicillium chrysogenum* into *S. cerevisiae*. The synthetic penicillin was reported to be effective against *Streptococcus* bacteria [23]. 

Synthetic biology has also been harnessed for environmental monitoring and biosensing, especially for heavy metals. In a report by Wang et al. [24], an AND genetic logic-gated biosensor in *E. coli* was used to sense arsenic, mercury, and copper. In a similar report, Wan et al. [25] engineered a multi-layer transcription factor for detecting arsenic and mercury up to 5000-fold and 750-fold, respectively. Synthetic biology has also been used to address challenging health problems. A notable example is the design of specific high-sensitivity enzymatic reporter unlocking (SHERLOCK), a CRISPR-based diagnostic tool for the fast and accurate detection of pathogens [26]. Gerber et al. [27] developed XNAzymes to target the spike-, nucleocapsid-, ORF1ab-, and ORF7b-encoding RNA of the SARS-CoV-2 virus in vitro and in vivo. In their in vivo methods, the XNAzymes acted as precise endonucleases, cleaving the SARS-CoV-2 RNA sequences, and reducing viral infection by about 75% in transfected cells [27]. 

These few examples show that synthetic biology can thus offer an important breakthrough in antiviral discovery, and in the ability to design new biologics and compounds that can degrade a viral genome before it takes over the host replication and translation machineries. 

In this review, we briefly discuss a mechanism of viral RNA degradation relevant to the design of genetic circuits, and report examples of genetic circuits designed to achieve the degradation of viral RNA within host cells. Finally, we give our perspectives on how natural RBPs can be used as ON/OFF switch circuits for viral RNA degradation. Although this review focuses on viruses, we envisage that this approach could be applied to any specific group of cellular RNAs.

## 2. Process of Viral RNA Degradation

All living organisms use RNA to produce proteins. In the case of viruses, the proteins are assembled to create new viral particles, which are needed for infecting other host cells. This is a controlled process through which the virus uses the host ribosomes’ translation machinery to make its proteins [3]. However, host cells have devised means to reduce viral load by breaking down the viral RNA’s non-specific host-defense immunity at the cellular level, thereby blocking the translation process. For instance, cellular exoribonuclease 1 (XRN1) plays a critical role in breaking down viral RNA, and it acts in the 5′-to-3′ direction [28,29]. It has been reported to work against various viruses [30]. The cleavage of viral RNA by XRN1 is essential for the host cell to survive a virus attack by regulating RNA turnover [31,32]. Despite the efficient activity of the cellular XRN1, many viruses contain folded RNA elements called exoribonuclease-resistant RNAs (xrRNAs) during the production of their protein-coding and noncoding subgenomic RNAs (sgRNAs) [33,34]. These xrRNAs act by blocking cellular 5′-to-3′ XRN1 endonuclease activity [32,33,35]. This mechanism is crucial to the survival of viruses, but presents a burden for host cells to processively degrade viral RNA.

RNA-binding proteins (RBPs) play a crucial role in the degradation of viral RNA. These proteins are characterized by their ability to bind specifically to RNA molecules, which can lead to changes in their function or in them being tagged for recognition by other cellular activities [36]. The binding of RBPs to RNA creates ribonucleoprotein (RNP) complexes that are essential for gene expression [37]. The interaction of RBPs with RNA can affect various aspects of RNA processing, including stabilization [38], localization [39], translation [40], and degradation [5]. For example, polyadenylating enzymes and poly(A) polymerase are first recruited to the mRNA in the nucleus to attach poly(A) to the 3′ end of the mRNA [41]. Then, the RBP-mRNA complex at the 3′ end may recruit other proteins that could assist in translation initiation or suppress translation [42]. poly(A)-binding proteins (PABPs) are known RBPs that interact with other cellular scaffold proteins such as eIF4G to stabilize the mRNA sequence before enabling translation [43]. 

**Figure 1:**
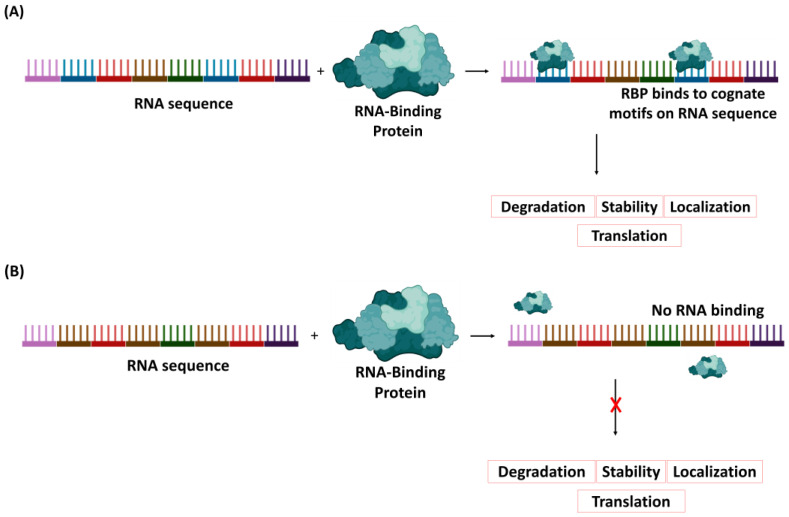
RNA-binding protein recognizing RNA sequence for processing. Different binding motifs on the viral RNA sequence are represented with different colors. (**A**) The RNA-binding protein recognizes the cognate motifs (blue) and facilitates further steps in the RNA processing either for degradation, translation, localization, or stability. (**B**) RNA without the recognizable blue motifs prevents binding of the RBP to the RNA, thereby truncating RNA processing.

RBPs recognize short nucleotide sequences, called motifs, on RNAs using RNA-binding domains (RBDs). These motifs are typically 4–10 base pairs in length and can be arranged in various configurations to create versatile binding positions [44]. Therefore, the interaction between the RBD and the motif on RNA is a crucial factor for RNA processing. When there is no recognizable motif on the RNA, there is a deficiency in protein–RNA interaction, which limits RNA processing (Figure 1). There are thousands of RBPs in various organisms, and while several hundreds of RBPs with RNA-binding domains have been identified, several RBPs still require further investigation [45].

Several RBPs have been identified in host cells to facilitate various viral processes. These RBPs are either expressed or upregulated by the host cells in response to the pathogen [46]. For host RNA, post-translational modification helps discriminate them from viral RNA, because mature host RNAs are first processed in the nucleus before being exported into the cytoplasm for translation, where they are labeled as “self” and protected from degradation. Studies have shown that some RBPs can influence viral replication [36]. However, several RBPs have also been reported to be involved in viral RNA degradation. For instance, zinc finger antiviral protein (ZAP), which is a host antiviral factor, binds to the CpG island of HIV-1 and directs its RNA degradation by interacting with cofactor KHNYN [47]; ZAP also binds to the enriched CG regions of SARS-CoV-2 RNA sequences to direct its degradation [48,49]. Kases et al. [50] demonstrated that ZC3H11A interacts with human adenovirus type 5 (HAdV-5) capsid mRNA, using zinc finger motifs (ZFM) to bind to the viral RNA in a PABPN1-dependent manner, whereas ZC3H11A with ZFM mutants showed reduced protein–RNA interaction [50]. Girardi et al. [51] have provided an extended review of the influence of RBPs on host–virus interaction [51]. 

Although the process takes place naturally inside a host cell after infection, attempts have been made to engineer RBPs to specifically target viral RNA for controlled degradation [44]. There are limited engineering studies on repurposing RBPs for viral RNA degradation, likely due to the lack of defined binding motifs. However, Laudenbach et al. [52] demonstrated the use of Nudix hydrolase 2 (NUDT2), a protein with high homology to bacterial RNA pyrophosphatase H (RppH), to cleave the viral 5′-triphosphate (PPP-) group to monophosphorylated (P)-RNA. The PPP-group has been shown to block the 5′-3′ canonical degradation pathway via XRN1 [53]. In editing dsRNA, Knight and Bass [54] showed that the RNA-editing enzyme ADARs can convert adenosine to inosine, which is then recognized as guanosine in the downstream process, resulting in a new sequence and RNA structure that are different from those of the wild type. Most studies use small drug molecules to activate the pathway for the engineered RBPs to act on the RNA [55]. Nevertheless, RBPs are becoming versatile tools to act as transcription activators or repressors in genetic circuits [56].

## 3. Design of Genetic Circuits

The principles of synthetic biology aim to achieve precise control over bio-inspired engineering [18,57]. This control enables the design of synthetic devices that can perform various tasks, such as producing valuable chemicals, designing biosensors, and developing nucleic acid-based therapeutics [58,59]. One of the critical aspects of this fascinating area of science is to create genetic or biological circuits that mimic electrical circuits to perform precise tasks [18,60].

**Figure 2:**
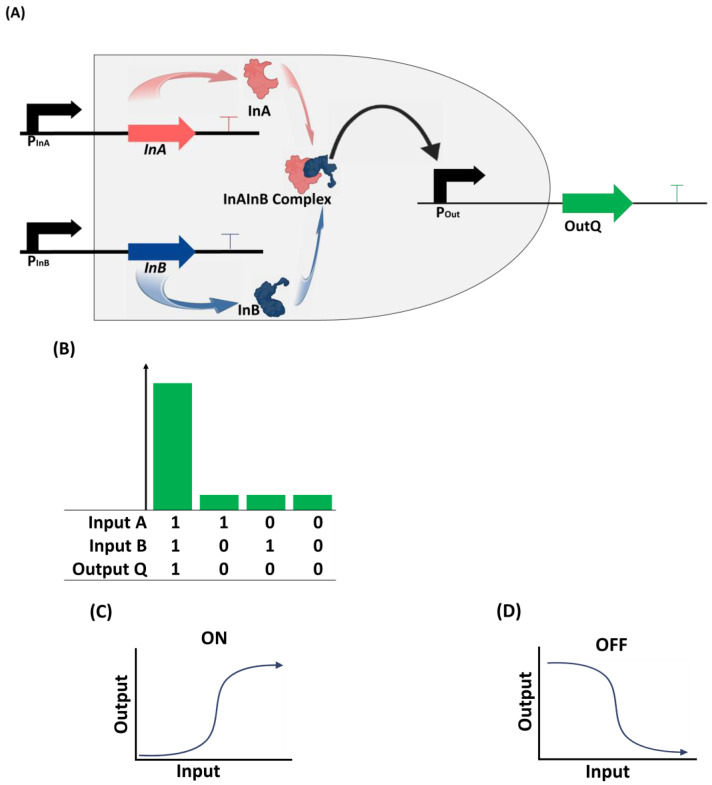
Principles of a simple genetic circuit design. (**A**) An AND logic gate with two promoters (PInA and PInB) for respective inputs (InA and InB). Expressed products (InA and InB) of both genes form a complex (InAInB Complex) that acts as a transcription activator to bind to the promoter (POut) of the output. The final product (OutQ) is predetermined in most cases to perform specific functions. The promoter regions are subject to fine-tuning to regulate the expression of the inputs or the output or both. (**B**) When both inputs A and B are expressed, the circuit is switched on and the expected output would be significantly expressed. The expression of either or none of the inputs would lead to low or no output. (**C**) When the circuit is switched on, the expected output increases significantly, and vice versa when switched off (**D**).

Genetic circuits are networks of genes and proteins that are designed to work together in a functional biological system for specific purposes [61,62]. These circuits are designed orthogonally so that the genes and proteins interact with each other for gene expression control, and they can control gene expression at the transcriptional or translational level [59]. Synthetic biologists often use a circuit design and construction model similar to that used in electrical engineering to design biological circuits. These synthetic circuits function similarly to living cells, producing input–output responses with defined characteristics such as biologics for theragnostic applications, enzymes and biomolecules for environmental sensing, or value-added chemicals for industrial applications [7,63,64,65,66,67]. These products are characterized by controlled signals designed in logic gates such as AND, OR, XOR, NAND, NOR, XNOR, and NOT (7). Figure 2 shows a simple genetic circuit design that uses an AND gate.

Since the first genetic circuit was designed in 2000 [68], scientists have developed new circuits with standardized biological parts to perform precise logic functions. Gene regulators, such as CRISPR/Cas [69,70], recombinases [71,72,73,74], RNA-binding proteins [75], and DNA-binding proteins [76], have been utilized in synthetic circuits. For example, CRISPR interference (CRISPRi) was used by Santos-Moreno et al. [77] to build three circuit designs: a synthetic oscillator (“CRISPRlator”), a bistable network (toggle switch), and a stripe pattern-forming incoherent feed-forward loop (IFFL). In the IFFL experimental design, a three-node fluorescent reporter (Nodes 1–3; N1–N3) was designed based on repression interactions. Their results showed that an increase in the expression of N1 suppressed N2 expression, while N3 peaked at the intercept of N1 and N2 expression, providing a monitoring system for the input concentration detector in the circuit, and spatial patterning [77]. Olorunniji et al. [73] designed a versatile split-intein serine integrase-based system with potential applications for synthetic circuits and memory device development. They used an AND gate for the split-intein system to express ϕC31 integrase to precisely control GFP and RFP production in *E. coli* under an invertible promoter via site-specific recombination (73). Wroblewska et al. [75] used RBPs to function as both the input and the output of RNA regulatory devices in post-transcriptional circuits, thus making it possible to design circuits that would have control over cellular behavior without genetic modifications. Numerous reports have further reviewed these biological parts and their functions within a given circuit [76,77,78,79,80,81,82,83].

The use of RNA circuit-triggering ON/OFF switches to target RNA for degradation is now a widely used functional approach in synthetic biology. In a recent study by Nakanishi and Saito, a transcription activator called Caliciviral VPg-based Translational activator (CaVT) was designed [56]. The device consisted of two proteins, an RBP MS2 coat protein (MS2CP) and a caliciviral VPg protein. The RBP MS2CP has a motif specific to its target RNA, while the VPg protein functions as a substitute 5′-cap structure. The CaVT could bind to the target RNA motif in the 5′ UTR direction and activate 5′ UTR translation without a canonical 5′-cap. The CaVT could regulate multiple mRNAs using a single protein and thus simultaneously activate and repress the translation of proapoptotic and antiapoptotic proteins in mammalian cells. It acts as a cell-fate regulator with RNA-only delivery. The RNA circuit was efficient in the activation and repression of Cas9 and anti-CRISPR AcrIIA4 translation, making it an essential tool in genome editing regulation [56].

## 4. Perspectives and Conclusions

**Figure 3:**
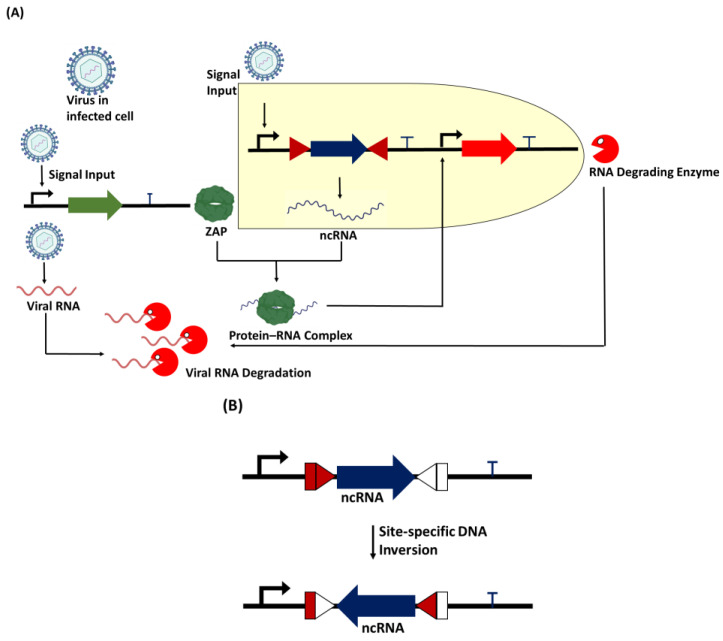
Proposed genetic circuit for viral RNA degradation. (**A**) When the virus infects a new cell, it triggers the expression of the upregulation of the RBP zinc finger antiviral protein (ZAP). The synthetic promoter in the circuit also allows the viral particles to trigger the expression of non-coding RNA (ncRNA) containing a sequence motif specific to protein. The protein–RNA complex acts as a secondary transcription activator to trigger the expression of the RNA-degrading enzyme, which is designed to specifically target the viral RNA in the host cell for degradation. (**B**) A recombinase such as serine integrase can act as a biological device to perform site-specific DNA inversion at specific att (attachment) sites to switch ON/OFF the expression of the non-coding RNA.

**Figure 4:**
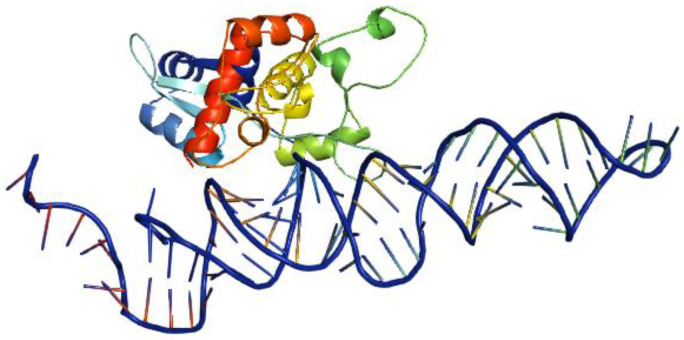
Zinc finger antiviral protein (ZAP) bound to an RNA molecule. Coordinates of the crystal structure of ZAP (PDB ID: 3U9G) were downloaded from www.rcsb.org, and HDOCK (http://hdock.phys.hust.edu.cn/, (accessed on 23 November 2023) was used to dock the RBP on the RNA with the 5′-CGUCGU-3′ binding motifs for ZAP [84]. The best model obtained was visualized using PyMOL version 2.5 [85].

While attempts have been made to use RBPs as transcription activators or repressors in genetic circuits to perform specific functions, there are fewer reports on the use of naturally upregulated RBPs inside an infected cell to direct the activation of RNA-degrading enzymes for viral RNA degradation. However, we have proposed a genetic circuit that would mimic the natural process of degrading viral RNA, but with a less complicated pathway (Figure 3). One of the limitations of this approach is the lack of a defined RBD-motif interaction between the protein and the vRNA. Many RBPs have multiple RBDs, which limits the precise design of RBD motifs in a genetic circuit. To control this, RNA sequences with defined singular motifs can be designed to bind to RBP with an RNA-binding domain specific to the RNA. For example, an RNA sequence with the optimal binding motif of ZAP C(n7)G(n)CG [84] can be designed to form a protein–RNA complex (Figure 4) that would serve as a secondary transcription activator for expressing degrading enzymes designed to target viral RNAs, including xrRNAs. Also, biological parts such as integrases can be incorporated as an ON/OFF switch to perform site-specific DNA inversion to regulate non-coding RNA binding to the ZAP (Figure 3).

We have proposed a hypothetical template for designing a genetic circuit that can degrade viral RNA by using natural host RBPs as part of the biological devices in the circuit. However, experimental methods are required to gain more insights into how this approach can be achieved. Key issues that need to be addressed include the following:

(1) Would the RBP of interest be upregulated during viral infection? This can be addressed by proteomic analysis of the infected cells. In addition, techniques such as quantitative proteomics, UV protein–RNA crosslinking, and the oligo(dT) selection of polyadenylated (poly(A)) RNA can be used to study expression patterns [86,87,88].
(2) If the RBP is upregulated, would the designed short non-coding RNA bind to the protein, and if it does, would the complex induce the expression of an RNA-degrading enzyme? Although the protein could be abundant in the cell and could bind to other RNAs as part of its cellular function, the careful design of the ncRNA with a motif specific to the protein is expected to enable the RNA to form a complex with some of the proteins, thereby triggering the expression of the RNA-degrading enzyme.
(3) How would the degrading enzyme recognize the RNA? This can be investigated by using specific methods such as the use of guide RNA to direct the enzyme to its target RNA cut site.
(4) How would we regulate gene expression in the circuit? The ncRNA is an important device in the circuit. The template provides a recombinase ON/OFF switch for the expression of the ncRNA by flipping its coding gene in the opposite direction. The recombinase can also be used to flip either the promoter or the terminator to control ncRNA expression.

The overall goal of designing this genetic circuit is to take advantage of the natural process of RBP activity inside the host cell for vRNA degradation. The operation of the proposed genetic circuit for targeted viral RNA degradation will be tested via a combination of mathematical modeling and experimental validation [89,90].
